# Metabolic and Molecular Mechanisms of Diet and Physical Exercise in the Management of Polycystic Ovarian Syndrome

**DOI:** 10.3390/biomedicines10061305

**Published:** 2022-06-02

**Authors:** Giorgia Scarfò, Simona Daniele, Jonathan Fusi, Marco Gesi, Claudia Martini, Ferdinando Franzoni, Vito Cela, Paolo Giovanni Artini

**Affiliations:** 1 Division of General Medicine, Department of Clinical and Experimental Medicine, University of Pisa, 56126 Pisa, Italy; g.scarfo1@studenti.unipi.it (G.S.); jonathan.fusi@med.unipi.it (J.F.); ferdinando.franzoni@unipi.it (F.F.); 2 Department of Pharmacy, University of Pisa, 56126 Pisa, Italy; claudia.martini@unipi.it; 3 Department of Translational Research and New Technologies in Medicine and Surgery, University of Pisa, 56126 Pisa, Italy; marco.gesi@unipi.it; 4 Division of Gynecology and Obstetrics, Department of Clinical and Experimental Medicine, University of Pisa, 56126 Pisa, Italy; celav2001@gmail.com

**Keywords:** polycystic ovarian syndrome, diet, physical exercise, epigenetic modifications

## Abstract

Polycystic ovary syndrome (PCOS) is an endocrine systemic disorder mainly characterized by a hormonal and metabolic disbalance that leads to oligo/anovulation, hyperandrogenism and the formation of ovarian cysts. Despite the progress that has been reached in its diagnosis and management, little is known about the molecular mechanisms and signaling pathways underlying the pathogenic mechanisms. In this sense, recent research has suggested that the influence of multiple factors, including age, environment, lifestyle and the disease state environment can change the clinical presentation of PCOS via epigenetic modifications. Variants in the genes encoding for proteins involved in steroidogenesis and glucose homeostasis play a crucial role in the development of the disease. Other genes involved in inflammation and cell proliferation seem to undergo an epigenetic control. Moreover, lifestyle factors influence the PCOS course and prognosis, including diet and physical activity, which are fundamental in reducing oxidative stress, inflammation and in improving metabolic and hormonal parameters. In the present review, literature evidence on molecular and epigenetic mechanisms related to PCOS etiology will be discussed, with a particular attention on the positive influence of diet and physical activity as nonpharmacological ways of intervention in the management of the disease.

## 1. Polycystic Ovary Syndrome: Definition and Pathogenic Mechanisms

Polycystic ovary syndrome (PCOS) is an endocrine, systemic disorder that affects 5–20% of women in reproductive age and is mainly characterized by a sex hormone imbalance causing the formation of water-retained cysts in the ovarian antral follicles [1]. The hormonal set is characterized by high luteinizing hormone (LH) levels and an elevated production of androgens, with the consequent virilization of female tracts and anovulation/oligovulation [2]. This hyperandrogenism and chronic anovulation are typical of PCOS, and related PCOS phenotypes mirror a self-powering cycle that involves metabolic and endocrine disorders [3]. The excess of androgens causes hyperinsulinemia, insulin resistance and obesity, which in turn negatively affect ovarian function and the hormonal profile [4]. Abnormalities in levels of gonadotropin-releasing hormone and follicle-stimulating hormone (FSH) are also present [5].

It has to be considered that about 75% of PCOS women suffers from anovulatory infertility [6], and those who undergo in vitro fertilization (IVF) have a higher risk of developing ovarian hyperstimulation syndrome. Moreover, the same women present a significantly higher risk of miscarriage and gestational diabetes during pregnancy [7].

Currently, the most used diagnostic criteria are the Rotterdam criteria (2003). According to them, PCOS is present if two of the following three criteria are manifested: oligo/anovulation, hyperandrogenism and the presence of ovarian cysts. Following the same criteria, four phenotypes could be determined [8]:

- oligo/anovulation + hyperandrogenism + polycystic ovaries;
- oligo/anovulation + hyperandrogenism;
- hyperandrogenism + polycystic ovaries;
- oligo/anovulation + polycystic ovaries [8].

Despite these well-characterized phenotypes, the pathogenesis of PCOS remains unclear; it is a multifactorial disorder that involves genetic, epigenetic and environmental variables [9], as well as immunity disorders [10].

**Figure 1:**
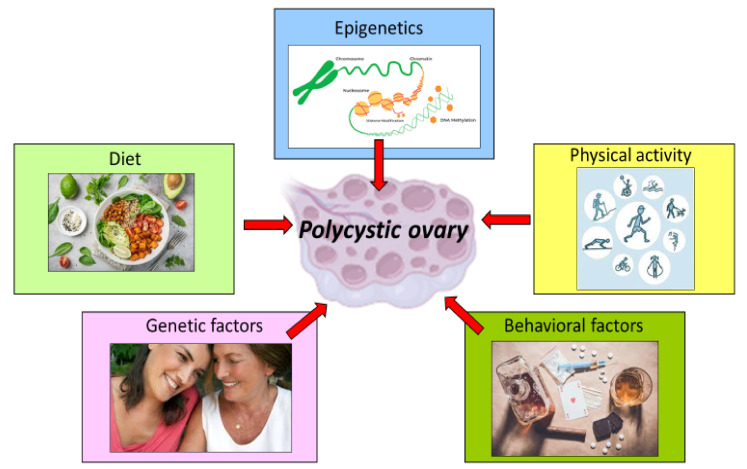
Genetic, environmental and behavioral factors involved in the pathogenesis of PCOS.

Moreover, lifestyle factors influence the PCOS course and prognosis. Despite the progress that has been reached in its diagnosis and management, little is known about the molecular mechanisms and signaling pathways underlying the pathogenic mechanisms. In this sense, recent research has suggested that the influence of multiple factors, including age, environment, lifestyle and the disease state environment, can change the clinical presentation of PCOS via epigenetic modifications. In the present review, the molecular and epigenetic mechanisms of diet and physical activity are discussed, with a particular attention on their positive influence in the management of the disease and their efficacy as nonpharmacological ways of intervention (Figure 1).

## 2. Mechanisms Involved in PCOS Pathogenesis

### 2.1. Molecular Mechanisms 

Dysfunctional granulosa cells (GCs), characterized by a poor apoptosis and increased proliferation, have proved to be typical of PCOS and are usually associated with impaired ovulation and folliculogenesis [11]. Elevated levels of circulating insulin could be responsible for GC abnormalities: specifically, it is able to favor the overexpression of mir-93 and the downregulation of mir-145, which in turn enhance cell growth and inhibit apoptosis [12,13]. 

Dysfunctional GCs also present increased mitochondrial autophagy that leads to mitochondrial injuries with consequent decreased membrane potential and mtDNA content [14]. More generally, elevated levels of androgens in PCOS women causes an overactivation of mitochondria in several tissues within the body favoring an ATP surplus [15]. An excessive amount of ATP molecules acts both on pancreatic β-cells and α-cells causing hyperinsulinemia and insulin resistance through the inhibition of the AMPK signaling pathway in insulin-sensitive tissues [16]. Insulin and insulin growth factor 1 (IGF-1) trigger some pathways in inner theca cells, directly causing its hypertrophy and the overproduction of androgens [17]. Particularly, they activate CYP17A1 and the steroidogenic factor (SF-1), favoring the adrenal steroidogenesis as well as the ovarian one [9]. In addition, hyperinsulinemia and hyperandrogenism affect pituitary LH pulsatility and worsen the release of male hormones [18].

The excess of androgens is directly related to the arrest in the antral follicle development and anovulation: in murine models, androgens have proved to enhance the activity of the C-type natriuretic peptide (CNP) and natriuretic peptide receptor 2 (CNP/NPR2) pathway that in turn stops oocyte meiosis impairing ovulation [19].

Androgens also correlate with GCs dysfunction, favoring in them the accumulation of advanced glycation end products (AGEs): male hormones, in fact, cause the overexpression of the AGE receptor in GCs through the activation of the endoplasmic reticulum stress [20]. In their turn, AGEs impair insulin signaling in GCs, affecting follicle growth [21].

Moreover, GCs undergo the epithelial mesenchymal transition (EMT) during the ovulation period, and this process is regulated by the integrin-interacting protein kindlin 2; it has been demonstrated that an excess of testosterone in PCOS women significantly increased the expression of kindlin 2, thus affecting EMT and ovulation [22]. In addition, EMT has proved to be linked to pathological processes within the endometrium of women suffering from PCOS, causing endometrial dysfunction and reproductive disorders [23].

In this kind of patient, a strict correlation between hyperandrogenism and abdominal adiposity has been highlighted as well. An in vitro study showed that in normal-weight patients, subcutaneous abdominal stem cells developed too early to adipocyte: these new adipose cells were characterized by a high content of lipids and an overexpression of PPARγ and CEBPα, which are two genes involved in adipogenesis processes [24]. In addition, these modifications were positively correlated with higher circulating levels of androgens [24].

### 2.2. Gene Polymorphisms

The genetic predisposition in PCOS is unquestionable and several genes are demonstrated to be involved in the etiology of the disease [25]. Indeed, due to the multifactorial nature of this disease, and to its polygenic contributions, it is not easy to offer a complete report about the genetic basis of PCOS [26]. Moreover, the different PCOS phenotypes surely complicate the genomic dissection of PCOS etiology. Most of genes involved in ovary functioning are potentially implicated in PCOS etiology, also considering the presence of an endocrine dysregulation and of inflammatory processes [26]. In this sense, the different gene types are often classified in subgroups based on their involvement in secretion and hormone functioning, as summarized in Table 1. With these premises, herein we provide the main genetic studies, also offering a connection with the pathological traits of PCOS (Table 1).

Considering that most PCOS women are at an increased risk of developing glucose intolerance and type 2 diabetes mellitus (T2DM), the gene Calpain 10 (CAPN10), located on the 2q chromosome and usually associated with insulin resistance, has been investigated [40]. In particular, UCSNP-44 polymorphism of the CAPN10 gene seems to have a remarkable role in the onset of the disease [40], while the UCSNP-43 genotype has been related to a worse metabolic profile in PCOS women [41]. Moreover, insulin resistance can be secondary to abnormalities involving insulin receptors, which are members of the tyrosine kinase family and are encoded by the INSR gene (chromosome 19p13.2) [42]. Notably, INSR rs1799817 polymorphism has been found to favor a worse glycemic pattern, as well as to predispose PCOS women to obesity [43]. In this sense, the fat mass and obesity-associated (FTO) gene plays a crucial role in developing PCOS: a recent study showed that the rs1421085, rs17817449 and rs8050136 variants of the FTO gene positively correlate with higher androgens levels and with an obese phenotype [44].

Critical genes that encode for steroidogenic enzymes, including cytochrome P450 enzymes (CYP), have also been investigated. In this regard, polymorphisms in CYP11A, CYP17, CYP19 and CYP21 genes are related to the alteration in the steroidogenesis with an overproduction of androgen hormones [45]. Recent studies have demonstrated that CYP 17 5’-UTR MspA1 (rs743572) and CYP 19 (rs2414096) polymorphisms increase the susceptibility to develop PCOS in young women, altering different hormone genesis pathways [46,47,48].

Since androgen effects are mediated by their receptors, it has been assumed that the androgen receptor (AR) gene is involved in hormonal disbalance. The AR, located on the X-chromosome at Xq11–12, presents a genetic polymorphism in exon 1, defined by a poly-glutamine (CAG) repeat region (8–35 repeats in most cases) [49]: a shorter length of this region has been typically associated with an increased androgen sensitivity; for this reason, CAG repeat polymorphism in the AR gene has been linked to PCOS amplifying women’s response to male hormones and leading to virilization and menstrual disorders [50].

Moreover, polymorphisms that occur in the sex hormone–binding globulin (SHBG) gene (chromosome 17p12—p13) seem to be crucial: it has been shown that a shorter (TAAAA) pentanucleotide repeat is responsible for an altered gene transcription [51]. As a consequence, poor circulating levels of SHBG cause obesity, impaired lipid metabolism, hyperinsulinemia, hyperandrogenism and chronic inflammation in women affected by PCOS [52].

In addition, reproductive anomalies in PCOS suggest the presence of mutations in folliculogenesis-related genes: for example, a recent study demonstrated that luteinizing hormone (LH) and luteinizing hormone receptor (LHR) genes show variants strongly related to PCOS phenotypes [53]. The FSHR (follicle-stimulating hormone receptor) gene also showed polymorphisms in PCOS women: interestingly, the two detected missense mutations, p.Ala307Thr and p.Asn680Ser, negatively affect ovarian FSH response, causing an impaired oocyte maturation, anovulation and consequent infertility [54]. A summary of the reported genetic polymorphisms associated to PCOS is reported in Table 2.

Despite the difficulties to offer a complete report about the genetic basis of PCOS, common genetic risk factors can be identified and linked to metabolic and endocrine dysfunctions associated to PCOS. Next generation genetic analysis will allow incorporating huge, genotyped datasets, thereby detecting a genetic architecture across PCOS diagnostic categories.

### 2.3. Epigenetic Mechanisms

Epigenetic mechanisms have been recently supposed to participate in PCOS pathogenesis: in particular, a huge amount of data is demonstrating that DNA methylation and microRNAs (miRNAs) have an altered pattern in the blood, serum, adipose tissue, granulose cells and theca of women with PCOS. Thus, PCOS women can have a different epigenetic regulation that seems to trigger and support the progression of the disease [56], possibly regulated by environmental elements, including diet and/or obesity and an adverse intrauterine environment [56]. Herein, we provide a summary of the main evidence related to DNA methylation, miRNAs and chromatin remodeling, connecting these alterations to protein functioning and PCOS pathological traits.

#### 2.3.1. DNA Methylation 

DNA methylation consists in an enzymatic reaction that adds a methyl group generally at the carbon in the 5′ position of the pyrimidine ring of a cytosine followed by a guanine called CpG dinucleotides [57]. A huge amount of data reveals that women with PCOS have an altered epigenetic program in part due to this epigenetic modification. Modifications in DNA methylation have been evidenced in tissues involved in PCOS pathogenesis, including ovary, adipose tissue and skeletal muscle [58], but also in umbilical cord blood, suggesting a correlation between the PCOS phenotype and epigenetic changes in cells from systemic and fetal circulation [58].

Furthermore, recent studies have highlighted that the mechanisms of DNA methylation possess a high plasticity across age and DNA methylation, and gene expression and the corresponding phenotype can be modified through therapeutic intervention in animal models, thus opening the possibility of therapeutic interventions in PCOS women as well [58,59,60].

Table 3 summarizes the main studies investigating DNA methylation variations related in PCOS women/animals, with changes in the gene expression and correlations between DNA methylation and PCOS clinical findings.

A general DNA methylome profiling of GCs recently has revealed an altered methylation in genes regulating pivotal ovarian functions in PCOS [66]: in particular, few differentially methylated genes, including aldo-keto reductase family 1 member C3, calcium-sensing receptor, growth hormone-releasing hormone receptor and tumor necrosis factor, which predominantly contribute to hyperandrogenism, premature luteolysis and oocyte development defects, can be explored as novel epigenetic candidates in mediating ovarian dysfunction. Overall, this study evidenced that the epigenetic dysregulation of genes involved in important processes associated with follicular development may contribute to ovarian defects observed in women with PCOS.

Several studies investigated the genome-wide DNA methylation levels in the peripheral blood of women with PCOS (for a detailed review, see [58]). Among these findings, Shen and collaborators have shown up to 40 genes with a different pattern of methylation in PCOS samples, mainly correlating with cancer, immune response, transcription regulation and metabolism [67]. Another study has identified additional genes with altered DNA methylation that correlate with inflammation and altered metabolism [68]. Particularly, in this study DNA methylation of specific CpG sites has been shown to be related to pathological clinical parameters in PCOS women (i.e., circulating estradiol and prolactin). In parallel to investigations related to genome-wide DNA methylation, other studies have focused on the DNA methylation pattern of specific genes [62,69,70,71], as reported in Table 3. Overall, the reported changes in DNA methylation are related with the molecular pathways and physiological processes that are dysregulated in PCOS, including follicular development [72], infertility [73], steroidogenesis [74], glucose metabolism and insulin signaling [75]. Of note, the investigation of DNA methylation in blood does not automatically reproduce the status of specific organs or tissues implicated by the disease [58]. Moreover, in these studies, DNA is isolated from different cell types: as a consequence, the methylation profile can be the average of the methylation levels of each cell type [76].

The molecular and morphological changes in the ovarian tissue that are typical of PCOS can be related to epigenetic alterations, as demonstrated in animal models of PCOS.

For example, the fetal programming induced by prenatal testosterone has been proven to be related with defects in the expression of ovarian steroidogenic genes correlated with the insulin pathway in the ovaries of fetal ewes [77]. Similarly, DHEA administration in prepubertal female mice resulted in a decrease of DNA methylation in oocytes [78]. DNA methylation changes in single oocytes has been related with transcriptional regulation and cell division [79]. Using dihydrotestosterone (DHT)-induced prenatally androgenized (PNA) mice as a model of PCOS, 857 differentially methylated genes and 3317 differentially expressed ones have been found compared to control mice [63]. In murine models, DNA hypomethylation regulates the expression of several key genes for developing PCOS; the same genes, involved in DNA demethylation (*TET1*), axon guidance (*ROBO-1*), inhibition of cell proliferation (*CDKN1A*), inflammation (*HDC*) and insulin signaling (*IGFBPL1*, *IRS4*), are found to be hypomethylated in PCOS women as well, and it has been suggested that this epigenetic process could be inheritable, since three of them (*ROBO-1*, *HDC*, *IGFBPL1*) resulted in being hypomethylated in daughters suffering from PCOS [65].

A few studies have stated significant differences in the methylated genes of the ovarian tissue between PCOS women and controls, which correlate with hormone activity (such as AMH), transcriptional regulation, inflammation, glucose metabolism and insulin signaling [71]. Furthermore, alterations in DNA methylation in PCOS women have been proven for different genes associated with the ovary function, and these modifications are related to a dysregulated response to gonadotropins, insulin signaling and steroidogenesis [64,71,80,81]. Similarly, significantly lower levels of DNA methylation in the LHCGR gene promoter with an increase in the gene expression have been reported in granulosa cells in PCOS women [71].

The epigenetic regulation affects also the Traf2- and Nck-interacting kinase (*TNIK*) usually involved in cell proliferation: the hypermethylation of the corresponding gene upregulates TNIK in PCOS ovarian tissue; moreover, the hyperexpression of TNIK seems to be related to Wnt signaling pathways, finally altering women metabolic profile [61].

In addition, epigenetic mechanisms correlate with the androgen excess. The hypermethylation of tumor necrosis factor (*TNF*) and the hypomethylation of aldo-keto reductase 1 C3 (*AKR1C3*), calcium-sensing receptor (*CASR*), growth hormone-releasing hormone receptor (*GHRHR*), resistin (*RETN*) and mastermind-like domain 1 (*MAMLD1*) lead to hyperandrogenism, causing the hormone disbalance that is crucial for the pathogenesis of PCOS [66]. Interestingly, the hyperandrogenic profile differs between PCOS obese and nonobese women, and the discrepancy occurs under the influence of an epigenetic control: in particular, the gene that encode for luteinizing hormone receptor *(LHR)* is overexpressed in PCOS nonobese women; in contrast, the insulin receptor gene *(INSR)* in under expressed in PCOS obese subjects [82]. Hyperandrogenism in PCOS women can be attributed to a decrease in the DNA methylation of the NCOR1 promoter (a nuclear corepressor of PPARG pivotal in reproductive functions and hormonal signaling) and an increase in the PPARGpromoter in granulosa cells (encoding the PPARG that regulates ovarian function) [64].

Moreover, considering that ovarian autophagy is critical for follicular development [83], the genes involved in these mechanisms were investigated. In PNA mice, a global hypomethylation is detected, resulting in an overexpression of genes involved in the mitogen-activated protein kinase (MAPK)/p53 pathway (*Mapk14, Mapkapk3 and Trp53*) and autophagy (Becn1) [63]. Interestingly, it has been found that the prenatal exposure to DHT (used in this model) is responsible for the hypomethylation, suggesting that hormonal changes influence the epigenetic control [63].

#### 2.3.2. MicroRNA

Together with the DNA methylation, microRNAs (miRNAs), which are small noncoding RNAs, are involved in epigenetic processes regulating gene expression at the post-transcriptional level [84]. The interaction between DNA methylation and miRNAs seems to have an important role in the pathogenesis of PCOS, since the hypermethylation of some miRNA promoter regions (miR-429, miR-141-3p and miR-126-3p′) upregulate the expression of genes controlling cell proliferation, inflammation and apoptosis *(XIAP, BRD3, MAPK14 and SLC7A5)* [85]. Actually, up to 30 miRNAs are differentially expressed in the PCOS women compared to controls, and are implicated in controlling ovulation, follicles and hormonal levels [86].

Altered miRNA expressions are closely associated with the occurrence of diseases, including endocrine and metabolic disorders. Recently, the roles of miRNAs in PCOS pathology have attracted considerable attention [87]. For example, the expression level of three miRNAs (miR-222, miR-146a and miR-30c) has been demonstrated to be significantly increased in PCOS patients with respect to the controls [88]. In this paper, Liu and colleagues have proved that miR-222 is positively associated with serum insulin, while miR-146a is negatively associated with serum testosterone. Furthermore, bioinformatics analysis indicated that the selected miRNAs are mainly involved in the regulation of cell cycle, apoptosis and endocrine functioning and can represent noninvasive biomarkers for PCOS diagnosis [88].

In addition, different miRNAs have been linked to PCO pathogenesis and different studies have enlightened the importance of miRNAs in cumulus GCs for ovarian follicle development. For example, Cai and collaborators [12] have proved a significant downregulation of miR-145 in the isolated human GCs of PCOS women, which has been related to an increase of cell proliferation and DNA transcription by the activation of the insulin receptor substrate 1 (IRS1) and of the downstream MAPK/ERK signaling pathway [12]. The pharmacological modulation of miR-145 can thus represent a therapeutic strategy to resolve GCs dysfunction in PCOS [12]. Furthermore, human GCs have been found to present significant lower levels of miR-126-5p and miR-29a-5p, along with the overexpression of its target, the klotho protein, which has been suggested to regulate GC survival/apoptosis by modulating the insulin/IGF-1, Wnt1 and Akt signaling pathways [11,89].

Another miRNA involved in GC proliferation is miR-93, whose expression has been proven to be upregulated in PCOS ovarian samples. This miRNA seems to regulate folliculogenesis and GC proliferation via targeting CDKN1A and promoting G1/S transition [13]. An elevated insulin concentration has been supposed to trigger GC proliferation by inducing miR-93 expression and downregulating CDK1A [13].

Similarly, miR-221/miR-222 has been evidenced as downregulated in GCs of PCOS samples and to be related to GC decreased proliferation, probably as a consequence of an elevated androgen level and overexpression of p27/kip1 [90].

A few members of the miR-17-92 cluster (i.e., miR-92a and miR-92b) have been found to be downexpressed in the ovarian tissues of PCOS women [91]. These miRNAs are believed to target the insulin receptor substrate proteins 2 (IRS-2), contributing to the hyperinsulinemia status of PCOS patients [91]. Moreover, miR-92a can bind the GATA binding factor 6 (GATA6), which is involved in androgen release, thus linking the insulin and androgenic signaling pathways in PCOS pathogenesis [91].

Additionally, other miRNAs have been related to the serum concentration of key hormones or of their receptors: for example, Song and collaborators have evidenced a downregulation of serum miR-592, whose targets are represented by luteinizing hormone/chorionic gonadotropin receptor (LHCGR), which regulates follicle development, and by IGF-1 receptor (IGF1R), which has been linked to insulin resistance [92].

#### 2.3.3. Chromatin Modifications

A few studies have studied the putative differences in the chromatin accessibility between PCOS women and controls, examining ovary samples but also samples derived from adipose tissue. For example, Hosseini and colleagues have investigated histone acetylation and methylation levels of cumulus cells (CCs) in infertile PCOS patients, and their correlation with the expression of the ovarian aromatase gene [93]. This study has evidenced a significantly higher incorporation of the histone H3K9ac mark in three analyzed promoters of CYP19A1 in PCOS than in the control group, highlighting an epigenetic contribution to aromatase gene expression in PCOS patients during controlled ovarian stimulation. In contrast, DNA methylation promoters and the histone H3K9me2 levels in promoter PII have been demonstrated to be significantly lower in the CCs of PCOS than those of the control group, suggesting a further epigenetic control of aromatase transcription during ovarian stimulation in PCOS patients [93].

A recent interesting study has evidenced that adipose stem cells (ASCs) of normal-weight PCOS women present an augmented lipid accumulation accompanied by an overexpression of the key master regulator genes of adipogenesis required for adipocyte maintenance/differentiation and specific changes in chromatin accessibility and transcriptional regulation compared to control stem cells [94]. Interestingly, there was an extensive correspondence between increased chromatin accessibility and increased RNA expression of the key genes involved in adipocyte differentiation and function, and in the triacylglycerol synthesis functional group during adipogenesis. Thus, the dynamic chromatin remodeling occurring during adipogenesis in abdominal ASCs of normal-weight PCOS women may improve adipogenic gene expression, finally promoting a greater fat storage [94].

The data presented here below allow concluding that methylation in DNA and miRNAs (and in part modifications in the chromatin status) is altered in PCOS women in the blood, serum, adipose tissue, granulose and theca cells. Thus, women with PCOS present a changed epigenetic regulation, which might be triggered by an adverse intrauterine environment or by postnatal environmental elements, including diet and obesity. These epigenetic alterations can be considered as biomarkers for the diagnosis and treatment of PCOS patients.

### 2.4. Immune Mechanisms 

An autoimmune predisposition could be involved in PCOS pathogenesis [10]. The frequent association with thyroid autoimmune diseases suggests that the autoimmunity could be at the basis of the disease [95]. In this regard, in women suffering from both PCOS and Hashimoto’s thyroiditis, levels of anti-Mullerian hormone (AMH) are negatively correlated with antithyroid peroxidase antibody (anti-TPO) levels, suggesting a decreased ovarian reserve in patients presenting these autoantibodies [96,97].

It has been demonstrated that, in women affected by PCOS, CD4(+), CD25(+) and CD127(−/low) T regulatory cells present a decreased activity with a consequent impaired immunosuppression [98]. Moreover, nonorgan-specific autoantibodies are often found in these women: particularly, antinuclear antibodies (ANA) have proved to be higher in PCOS women and are associated to clinical signs of hyperandrogenism [99].

Antiendometrial antibodies were also found in PCOS women and they have been demonstrated to associate with the presence of oxidized proteins, suggesting a link between the pathogenesis of the disease and the oxidative stress in the uterine microenvironment [100]. Interestingly, in these patients, levels of IgM antibodies against phosphorylcholine (IgM anti-PC), which are natural antibodies protective against atherosclerosis, are found to be lower, predisposing PCOS women to cardiovascular diseases [101].

Recent studies showed the presence of activating autoantibodies (AAb) to the gonadotropin-releasing hormone receptor (GnRHR) [102,103] that seem to be associated with increased levels of testosterone and inflammatory cytokines [102]. The same antibodies, in murine models, are also responsible for increased levels of LH, TNF-α, IL-1α and IL-18, enhancing inflammation and hormonal disbalance [104].

Low levels of LH and FSRH autoantibodies were also found, but their role in enhancing hyperandrogenism in PCOS women is still doubtful [105].

## 3. Impact of Lifestyle and Behavior in the Management of PCOS

Improving nonpharmacological interventions is fundamental in the management of PCOS. Lifestyle changes aim to achieve better anthropometric parameters that usually reflect better metabolic and hormonal profiles affecting positively ovarian functions [106]. Obesity is considered one of the major risk factors of infertility in women suffering from PCOS [107]. In particular, the adipose tissue, considered as an endocrine organ, produces specific and nonspecific cytokines, including adiponectin (APN), resistin, visfatin, omentin, retinol binding protein-4 (RBP4), lipocalin-2 (LCN2), chemerin, interleukin 6 (IL6), interleukin 1β (IL1β) and tumor necrosis factor α (TNFα), that are able to affect negatively insulin resistance and hormonal release [108].

First of all, identifying and correcting abnormal eating disorders is a successful strategy in providing weight loss [106]. In fact, PCOS women are more susceptible to depressive disorders and altered eating behaviors [109]. Moreover, depression has proved to be a typical tract of PCOS that affects about 40% of these patients [110] and is responsible for a worse metabolic profile: depression, in fact, is considered an inflammatory disorder and the overlap of symptoms between PCOS and depression suggests that mood modifications would be desirable to achieve better PCOS phenotypes [111].

In order to improve the metabolic profile, it has to be considered that sleep duration is crucial in developing worse PCOS phenotypes: a recent study demonstrated that poor sleep is associated with sleep apnea disorders, which in turn are responsible for increased levels of triglycerides, a higher percentage of liver fat and increased waist circumferences in adolescents affected by PCOS [112]. The resulting intermittent hypoxia causes oxidative stress and mitochondrial dysfunction: free radicals are able to activate several kinases, including NK, p38 MAPK and IKK, that trigger pathways involved in the insulin sensitivity [113]. Moreover, in rodent models, the intermittent hypoxia leads to pancreatic apoptosis (via Caspase 3 pathways), causing insulin resistance [114]. In addition, hormones that regulate appetite and insulin resistance follow a circadian rhythm. The disruption of this rhythmicity alters the release of hormones like growth hormone (GH) and leptin; GH-elevated levels in wake periods impair glucose metabolism, while the lowest levels of leptin in sleep deficiency increase appetite [115]. Therefore, interventions aimed to improve sleep quality and quantity could ameliorate women’s metabolic status and contribute to weight loss in PCOS women.

In this perspective, it is clear that diet and physical activity are keystones to achieve lifestyle changes which are effective in obtaining the improvement both in the metabolic and in hormonal profile [116].

## 4. Dietary Modification

Quantitative and qualitative changes in dietary patterns are needed to have an impact on the management of PCOS [117]. Generally, it has been demonstrated that both ordinary hypocaloric diet and a high-protein/low-glycemic-load hypocaloric diet positively affect the insulin sensitivity and the hormonal disbalance, with a significant reduction of testosterone levels and increased estrogens ones [118]. A modest reduction in carbohydrate (CHO) intake affects β-cell responsiveness, reducing basal β-cell response, fasting insulin, fasting glucose, total testosterone and all cholesterol measures, with a clear positive effect on insulin resistance [119]. As well as improving the metabolic profile, dietary patterns characterized by a lower glycemic index positively influence ovulation, fertility and cardiovascular features in PCOS women [120]. In addition, a high fiber consumption is recommended: in fact, a poor fiber intake means a higher glycemic load that worsens the insulin resistance [121].

The effect of a ketogenic diet on PCOS outcomes has also been investigated. A ketogenic diet is usually characterized by high fat, moderate protein and very low (40–50 g/die) CHO intake [122]. Evidence suggests that this specific pattern is able to improve anthropometric and biochemical parameters (including LH, FSH, SHBG) and reduce abnormal estrogen production deriving from the aromatization of androgens that occur in the adipose tissue, with a consequent improvement of the LH/FSH ratio [122,123]. Considering that in PCOS anovulation and infertility are aggravated by a chronic inflammatory state that affects ovaries and the uterine cavity [124], the importance of a ketogenic diet lies also in its anti-inflammatory properties mediated by the ketone β-hydroxybutyrate (BHB). Indeed, in murine models, this metabolite seems able to block the activation of NLRP3 inflammasome that usually is responsible for IL-1β and IL-18 production in human cells [125].

In this regard, in PCOS women, oral supplementation with natural molecules has been surprisingly useful in reducing inflammation. It has been shown that a magnesium–zinc–calcium–vitamin D cosupplementation reduces oxidative stress and inflammation in women affected by PCOS, increasing antioxidant capacity [126]. This is reflected in a reduced plasma malondialdehyde (MDA), that is a marker of lipid peroxidation [127], and consequently a better metabolic profile [126]. Interestingly, magnesium and zinc supplementation for 12 weeks is even able to interfere with the expression of genes that encode for inflammatory cytokines (IL-1, TNF-α), besides reducing blood high-sensitivity C-reactive protein (hs-CRP), protein carbonyl and increasing antioxidant mechanisms [128]. Moreover, magnesium alone has a remarkable role in regulating glucose metabolism, since magnesium serum levels have been demonstrated to be related to an improved insulin resistance [129].

An enhanced glucose and lipid metabolism has been observed in PCOS women also during vitamin D and calcium supplementation that positively affects triglyceride, cholesterol, testosterone levels, dehydroepiandrosterone sulfate (DHEAS), SHBG and hs-CRP [130]. In addition, vitamin D has anti-inflammatory and antioxidant capacities which reflect in reduced hs-CRP and MDA levels [131]. Because of their well-known anti-inflammatory properties [132], the relation between omega-3 polyunsaturated fatty acids (n3 PUFAs) supplementation and PCOS has also been investigated. Evidence suggests that n3 PUFAs positively affects inflammation, oxidative stress and hormonal parameters, reducing hs-CRP, MDA, total testosterone and increasing antioxidant capacity [133], even though these effects seem to be limited to women over 40 years old and with cardiovascular and metabolic abnormalities [134]. Specifically, n3 PUFAs, including eicosapentaenoic acid (EPA) and docosahexaenoic acid (DHA), act on inflammation, reducing TNF-α, IL-6 and IL-1β, and interfering with the nuclear factor kappa B (NF-κB) activity, reducing the activation of several proinflammatory genes [135]. Moreover, n3 PUFAs favor the β-oxidation of mitochondrial fatty acids, reducing lipid deposits and free radical genesis [135].

The inositol supplementation seems to be particularly effective in PCOS women. Inositol is a hexahydroxy cyclohexane (six-carbon ring compound) whose two isoforms, myo-inositol (MI) and D-chiro-inositol (DCI), respectively mediate insulin uptake in the ovaries and insulin-dependent androgen production [136]. In particular, starting from animal models, it has been demonstrated that the supplementation that led to 40:1 MI/DCI plasma ratio enhanced insulin sensitivity, reduced testosterone levels and was able to restore ovulation in most cases [136,137]. However, despite the effectiveness on the metabolic profile, the role of the MI: DCI (40:1) treatment is controversial; in fact, it has been suggested that this specific formulation could be a first-line treatment in normal-weight PCOS patients without insulin resistance, since a recent study demonstrated that ovulation and spontaneous pregnancies occur mostly in nonobese PCOS women [138]. Despite this, it is clear that adequate doses of inositol inside cells are fundamental for a correct function of the tissues, and detecting the exact formulation of inositol for each patients (according to their phenotypes) appears crucial for restoring ovulation and improving fertility [139].

The ovarian microenvironment is also influenced by the oral supplementation with L-carnitine; in a murine model, acetyl and propionyl L-carnitine have proved to enhance ovarian functions though the amelioration of the ovarian oxidative stress status, preventing the upregulation of SIRT1, SIRT3 and SOD2. Moreover, the same study demonstrated that L-carnitine acts against glycative molecular pathways [140]. In PCOS women, L-carnitine is also able to improve the metabolic profile, with consequent better levels of insuline, adiponectin, testosterone, LH and FSH [141].

In addition, a nonpharmacological intervention with flavonoids should be considered in women suffering from PCOS because of their large anti-inflammatory and antioxidant activities [142]. In murine models, flavonoids extracted from Nervilia Fordii are able to positively balance FSH, LH, testosterone and insulin levels, preventing JAK2/STAT3 pathway activation and upregulating IL-6 expression [143].

Finally, it must be considered that a balanced diet modulates intestinal biodiversity that, in turn, has an impact on the PCOS phenotype. The gut microbiota refers to a group of nonpathogenic microorganisms with anti-inflammatory, antioxidant, immune and metabolic properties for which a relationship with several diseases, including PCOS, has been established [144]. In murine PCOS models, gut microbiota negatively affects levels of triglycerides, cholesterol, testosterone, TNFα and lipopolysaccharides, increasing the inflammatory status [145]. In general, in women suffering from PCOS, the increased abundance of *Escherichia and Shigella* leads to an altered production of short-chain fatty acids, while a lower abundance of *Lactobacillus* and *Bifidobacterium* species impairs the release of anti-inflammatory metabolites and reduces the immune response [146]. In this regard, dietary patterns that include large fiber consumption seem able to improve PCOS clinical manifestations just acting on gut microbiota; into specifics, a recent study has reported that a 12-week high-fiber diet, or in combination with Acarbose, favors *Lactobacillus* and *Bifidobacterium* abundance with a positive effect on LH/FSH ratio, testosterone, homeostasis model assessment-insulin resistance (HOMA-IR), α-1-acid glycoprotein (α-AGP) and leptin [147]. In murine PCPS models, dietary α-linolenic acid, which is an n3 PUFA, has been shown to modulate gut microbiota, increasing Lactobacillus and Bifidobacterium as well as Allobaculum, Butyrivibrio, Desulfovibrio, Faecalibacterium and Parabacteroides, with a consequent reduction of plasma and ovarian inflammatory cytokines, including IL-6,IL-1β, IL-10, IL-17A, TNFα and monocyte chemoattractant protein-1 [148].

Therefore, gut microbiota plays a crucial role in PCOS pathogenesis and the use of probiotics as modulator of intestinal microorganisms should be considered as an alternative therapeutic strategy.

## 5. Exercise Interventions in PCOS Women

In order to obtain significant lifestyle changes, and in addition to a balanced diet, physical activity is fundamental for improving both metabolic and hormonal parameters as well as reducing risk factors for cardiovascular diseases [149]. As shown in a recent study, distinct types of training (high-intensity interval training, HIIT and continuous aerobic exercise training, CAET) were equally capable to act both on anthropometric and fertility profiles. In particular, after 6 months of exercise, women underwent a significant reduction of BMI and insulin resistance and improved their cardiorespiratory fitness (VO2 max); most importantly, some of them had documented ovulation and achieved pregnancy after the protocol [150]. Another study showed that a 3 month structured training reduced the peak oxygen consumption, body mass index and C-reactive protein, improving the cardiopulmonary function and decreasing the cardiovascular risk [151].

Besides improving BMI and cardiorespiratory functions, aerobic exercise seems able to affect catecholamine-induced lipolysis in PCOS women. A 6-week aerobic training has proved effective in increasing in vitro atrial natriuretic peptide (ANP)/isoproterenol-lipolysis, enhancing the sensitivity to ANP and isoproterenol and the postreceptor response via cAMP-dependent protein kinases [152]. This could favor weight loss, which is crucial in the management of PCOS women. A low volume HIIT is also able to modulate the expression of factors involved in fatty acid metabolism and inflammation; specifically, PCOS women seem to have higher levels of miRNA-27b (c-miR-27b), which is associated with an altered lipid metabolism and inflammatory status: a 16-week low volume HIIT has proven to be effective in reducing levels of c-miR-27b, thus improving the patient metabolic profile [153].

Moreover, an aerobic protocol seems capable to reduce inflammation markers in women affected by PCOS: especially in obese women, the inflammatory status is predominant [154] and often characterized by higher circulating leukocytes. Sixteen weeks of an aerobic training are useful to improve the insulin resistance and decrease the white blood cell (WBC) count. These changes correlated with a reduced serum leptin and the ratio of leptin to high-molecular-weight adiponectin [155]. Regarding exercise effects on hormonal outcomes, in murine models, a 15-day aerobic training has proved to be useful in decreasing inflammation and restoring the hormonal balance acting on levels of leptin, testosterone, estradiol, FSH, AMH, TNF-α, IL-6 and free fatty acids (FFA) [156]. Exercise positively affects the hormonal profile also in women: both continuous and intermittent aerobic training have been demonstrated to be useful in reducing circulating levels of testosterone as well as obesity parameters [157]. Controversial is the role of strength training in PCOS management, since it seems to increase androgen levels [158]. However, after a 4-month strength training, PCOS women had reduced levels of testosterone and a lower testosterone/androstenedione (T/A) ratio [159].

Physical activity also affects mitochondria that have been shown to be involved in PCOS pathogenesis [160]. In PCOS women, the mitochondrial DNA copy number is lower compared to women not affected by PCOS, and several mtDNA mutations occur in response to the insulin resistance and hyperandrogenism which enhance the oxidative stress, causing mtDNA abnormalities [160]. Different kinds of training protocols have demonstrated that exercise improves mitochondrial biogenesis and respiration upregulating PGC-1α expression [161] that is crucial for the transcriptional control of mitochondrial functions [162], even though further studies are needed to evaluate the effective role of exercise on mitochondria in PCOS.

Certainly, physical activity is effective in improving antioxidant defenses. In PCOS women, a 12-week aerobic training has proved to increase superoxide dismutase (SOD) and the total antioxidant capacity, and reduce MDA and AMH [163].

Finally, the role of exercise-induced irisin should be considered. Irisin is a hormone-like myokine whose production in the skeletal muscle is mediated by physical activity and seems to be crucial in improving biochemical aspects of several metabolic diseases, including PCOS [164]. Exercise is responsible for an overexpression of PGC-1α that in turn activates *FNDC5* with the consequent release of irisin, which is also produced in response to ATP depletion after exercise [165]. The importance of this hormone-like myokine lies in its capacity to impact glucose homeostasis and reduce systemic inflammation [166]. Indeed, irisin is able to brown the white adipose tissue-activating *UCP1* gene and increase the expression of GLUT4 in the brown adipose tissue, leading to an enhanced glucose uptake; it is also involved in lipolysis mechanisms triggering the AMP (cAMP)–protein kinase A (PKA)–perilipin–hormone-sensitive lipase (HSL) pathway [165].

**Figure 2:**
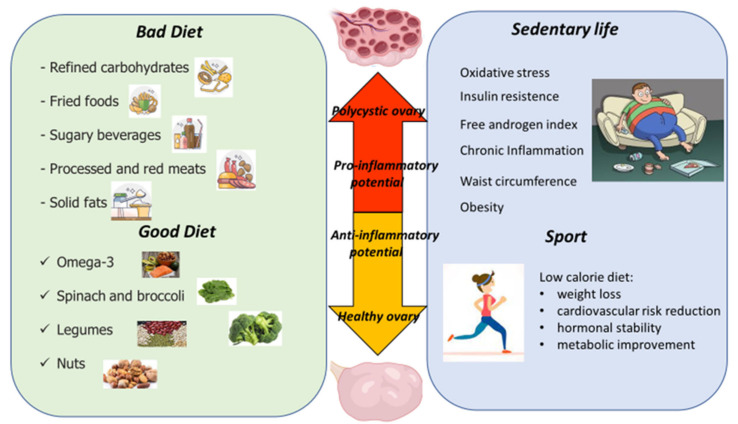
The influence of diet and physical activity on inflammation and ovarian health.

Because of its effect on inflammation, metabolism, endocrine and fertility parameters, physical activity should be always considered primary in the management of PCOS (Figure 2).

## 6. Conclusions

PCOS is a multifactorial disorder whose real pathogenesis is still unclear. Continuous interaction between genetic and environmental factors seems to influence different phenotypes of the disease through epigenetic and molecular mechanisms.

Differently from previous literature reviews in the field, the present work does not focus on a single topic, but it offers an overview on PCOS pathogenesis and nonpharmacological treatments, focusing on epigenetic mechanisms related to PCOS pathogenesis and on the reversal effects of diet and physical exercise. These approaches have a predominant role in triggering several pathways that affect PCOS clinical manifestations in a positive way. Next generation genetic analysis will allow incorporating huge, genotyped datasets, possibly generating genetic risk scores enhanced with clinical information, environment and lifestyle data for a precision medicine approach to PCOS diagnosis and treatment. Considering the above, more attention should be focused on these aspects to better improve the nonpharmacological strategies that appear fundamental in the management of PCOS.

## Figures and Tables

**Table 1:** Pivotal genes that have been involved in PCOS etiology.

Gene	Encoded Protein	Implication	Reference
*INSR*	Insuline receptor	**Insulin secretion and action**	[27]
*CAPN10*	Calpain 10 protein		[28]
*IRS1, IRS2*	Insulin receptor substrates IRS1 and IRS2		[29]
*FSHR*	Follicle-stimulating hormone receptor	**Gonadotropin release regulation**	[30]
*LH*	Beta subunit of luteinizing hormone		[31]
*AMH*	Anti-Müllerian hormone		[32]
*CYP21*	21-hydroxylase enzyme	**Synthesis pathways of steroid hormones**	[33]
*CYP11a*	A cytochrome P450 enzyme		[34]
*CYP19*	Cytochrome P450 aromatase	**Androgen synthesis pathways**	[35]
*CYP17*	Enzyme cytochrome P450-C17		[36,37]
*TNF-α*	The cytokine Tumor Necrosis Factor	**inflammation**	[38]
*SHBG*	Sex Hormone–Binding Globulin	**Regulation the androgen level in the body**	[39]

**Table 2:** Genetic polymorphisms associated to PCOS.

Gene	Polymorphisms	Effects on PCOS Phenotype
*Calpain 10 (CAPN10)*	UCSNP-44	PCOS pathogenesis [40]
	UCSNP-43	Worse metabolic profile [41]
*Insulin receptor gene (INSR)*	rs1799817	Worse glycemic pattern and obesity [42]
*Fat mass and obesity-associated gene (FTO)*	-rs1421085 -rs17817449 -rs8050136	High androgen levels [44]
*Cytochrome P450 enzymes (CYP):*		
*-CYP 17*	-rs743572	Steroidogenesis and alteration of hormonal pathways [46,47,48]
*-CYP 19*	-rs2414096	
*Androgen receptor gene (AR)*	poly-glutamine (CAG) repeat region	Amplified response to male hormones [50]
*Sex hormone–binding globulin gene (SHBG)*	short (TAAAA)n pentanucleotide repeat	Obesity, impaired lipid metabolism, hyperinsulinemia, hyperandrogenism and chronic inflammation [55]
*Follicle-stimulating hormone receptor* *(FSHR)*	-p.Ala307Thr -p.Asn680Ser	Impaired oocyte maturation, anovulation, infertility [54]

**Table 3:** Differentially methylated genes reported in tissues from patients with PCOS.

Genes	DNA Methylation Mechanism	Tissue	Clinical Effects Related to DNA Methylation Changes	References
*LY6G6F,* *KCTD21, ADCY9,* *RABL2B, ZNF611,* *VASH1, FST, LMNA, PPARGC1A*	Hypermethylation	Peripheral blood	Increased prolactin and estradiol levels in serum, increased free androgen index, insulin resistance, increased triglyceride levels in plasma and risk for metabolic syndrome	[61,62]
*L-1,* *TMSB15B, RPF1, DNA2,* *EPHA8, LHCGR* *EPHX1*	Hypomethylation			
*MATN4,* *DLGAP2, CDH13,* *GAREM2, GSC,* *ANKRD34C, ATP8B2* *PPARG*	Hypermethylation	Granulosa cells	Hyperandrogenism	[62,63,64]
*L-1, LHCGR,* *SMG6, CCR5, LHB,* *NTN1, ARFGAP1,* *MDGA1, NCOR1, YAP1,* *CD9, NR4A1, EDN2,* *BNIP3, LIF*	Hypomethylation			
*ZZEF1, TPT1,* *STUB1, DMAP1,* *RAB5B, PPARG, SVEP1,* *SAV1, RORA, RAB6A* *CNST*	Hypermethylation	Subcutaneous adipose tissue	Oligomenorrhea; Increased testosterone levels in circulation	[60]
*PUM1,* *DIP2C, SNX8, SRGAP3,* *ZFHX3, OR52W1and* *BBX*	Hypomethylation			
*TET1* *ROBO 1* *CDKN1A* *HDC* *IGFBPL1* *IRS4*	Hypomethylation	Ovarian tissue	Pathogenesis of PCOS	[65]
*TNIK*	Hypermethylation	Granulosa cells	Altered metabolic profile	[61]
*TNF*	Hypermethylation	Granulosa cells	Inflammation and hyperandrogenism	[66]
*AKR1C3* *CASR* *GHRHR* *RETN* *MAMLD1*	Hypomethylation	Granulosa cells	Hyperandrogenism and hormonal disbalance	[66]

## Data Availability

Not applicable.
